# Supplementary material for: A high-resolution mRNA expression time course of embryonic development in zebrafish
Source: eLife. 2017 Nov 16;6:e30860. doi: 10.7554/eLife.30860 (PMC5690287; doi:10.7554/eLife.30860)
Supplement: Supplementary file 6. [file elife-30860-supp6.zip › biolayout-clusters-files/Cluster135.html]

Cluster135


# Cluster135: Detail

### Go to ZFA detail

## GO

| GO ID | Description | Domain | Annotated | Expected | Observed | Adjusted p-value | Genes | Ensembl IDs |
| --- | --- | --- | --- | --- | --- | --- | --- | --- |
| GO:0000978 | RNA polymerase II core promoter proximal... | molecular\_function | 68 | 0.05 | 3 | 0.0021 | si:dkey-23a23.3 znf1135 znf1141 | ENSDARG00000088214 ENSDARG00000093424 ENSDARG00000102735 |
